# The impact of absent vulvar cancer screening guidelines on outcomes of vulvar squamous cell carcinoma: a national cancer database study

**DOI:** 10.1007/s10552-026-02141-4

**Published:** 2026-02-25

**Authors:** Grace Folino, Elizabeth Byrne, Mary Kate Eiden, Mya Vasa, Peter Silberstein, Marco DiBlasi

**Affiliations:** 1 Creighton University School of Medicine, Omaha, NE USA; 2 Department of Oncology, CHI Health, Omaha, NE USA

**Keywords:** Squamous cell carcinoma, Vulva, Vulvar cancer, Survivorship

## Abstract

**Purpose:**

To analyze current vulvar squamous cell carcinoma (VSCC) data with respect to age groups and determine if gynecologic cancer screening guidelines address the burden of VSCC on the ≥ 65 cohort.

**Methods:**

Patient data from 2004 to 2021 was identified from the National Cancer Database using ICD-10 codes specific for the vulva, and ICD-O-3 histology codes for squamous cell carcinoma or premalignant vulvar intraepithelial neoplasia Grade III. GraphPad Prism and IBM SPSS were used to analyze variable frequency with cross analysis. Chi-squared tests, Kaplan–Meier survival curves with log-rank comparison, and Cox proportional hazard regression models were utilized for statistical analysis. For regression models of hazard ratios (HRs) and odds ratios (ORs), the 50–64-year-old cohort was used as a reference variable.

**Results:**

The patient population was 68,153. Mean overall survival decreased as age increased (≤ 49 years old was 184.9 months, 50–64 years old was 152.1 months, 65–74 years old was 107.3 months, and ≥ 75 years old was 57.7 months). The ≥ 75-year-old cohort had a 330% higher risk of mortality when compared to the reference variable (HR 4.3, *p* < 0.001), followed by the 65–74-year-old cohort (HR 1.96, *p* < 0.001). The ≥ 75 years old and 65–74-year-old cohorts had the highest likelihood of advanced VSCC (OR 1.91, *p* < 0.001 and OR 1.37, *p* < 0.001, respectively).

**Conclusions:**

Patients ≥ 65 years old are significantly more likely to experience worse survival outcomes and higher stage diseases, indicating that a lack of screening protocols may influence VSCC outcomes.

## Introduction

Vulvar squamous cell carcinoma (VSCC) incidence has risen in the United States, with studies citing a 1.3% increase in incidence from 1999 to 2015 [1–4]. According to the American College of Obstetricians and Gynecologist (ACOG), prevention of vulvar cancer is limited to the HPV vaccine, and detection may be constrained to external genitalia examinations due to the absence of dedicated screening tests [5]. Clinicians often rely on cervical cancer screening visits to identify concerning or abnormal lesions on the vulva. However, routine cervical cancer screening is discontinued at age 65 with adequate prior negative results, potentially creating a gap in the detection of vulvar cancer in older patients.

Given the increasing incidence of VSCC and the aging population of the United States, it is important to reexamine the data to determine if further recommendations may be made. One previous study analyzed the data from the surveillance, epidemiology, and end results (SEER) database to assess vulvar cancer trends from 1992 to 2014 and emphasized the importance of simple genital examinations for early cancer detection [6]. This analysis will utilize the most up-to-date data from the National Cancer Database (NCDB) to examine age and survival trends in the context of current gynecologic screening guidelines. The aim of this study is to analyze the data provided by the NCDB to examine if current guidelines adequately cover the older gynecologic patient population affected disproportionately by VSCC.

## Methods

The American College of Surgeons (ACS) and the American Cancer Society (ACS) jointly sponsor the NCDB, a clinical oncology database providing data from hospitals accredited by the Commission on Cancer [7]. The Vulvar Participant User File provided by the NCDB contained anonymized patient data from 2004 to 2021. Following as previously described in the literature, patient data was identified using ICD-10 diagnosis codes, including malignant neoplasm of the labium majus (C51.0), malignant neoplasm of the labium minus (C51.1), malignant neoplasm of the clitoris (C51.2), malignant neoplasm of overlapping sites of the vulva (C51.8), and unspecified malignant neoplasm of the vulva (C51.9). Furthermore, the data were filtered for ICD-O-3 histology codes to identify the squamous cell carcinoma: papillary cell carcinoma (8052/2 and 8052/3), squamous cell carcinoma (8070/2–8076/3, 8078/3–8084/3), and squamous intraepithelial neoplasia of grades III or high grade (8077/2). All other primary sites and histology codes were excluded from data analysis.

Age at diagnosis was divided into the following cohorts: < 50 years old, 50–64 years old, 65–74 years old, and ≥ 75 years old. Age cohorts were separated in this fashion to create a cohort within the age of cervical cancer screening (50–64 years old) and outside of the age of cervical cancer screening (65–74 years old). In doing so, the *within screening* 50–65-year-old cohort was utilized as a reference variable for further analysis.

Demographic frequencies included age as divided into the cohorts described, race, and ethnicity. Race was defined as White, Black, American Indian, Asian, or Unknown. Ethnicity was categorized as Non-Hispanic, Hispanic, or Unknown.

Using NCDB’s all-cause mortality survival data, Kaplan–Meier survival curves were utilized to evaluate mean survival time based on age cohort and tested via log-rank pairwise comparisons. Cox regression multivariate analysis was performed to calculate the proportional hazard ratios of the four age cohorts. Next, the odds of having advanced VSCC and odds of undergoing surgical intervention for VSCC were calculated via binomial regression. Localized VSCC was determined to be stages 0–I, representing VSCC within the vulva or perineum without spread to lymph nodes. Advanced VSCC was determined to be stages II–IV, representing VSCC that has extended beyond the vulva or perineum or has spread to a lymph node. Surgeries that were assessed include local tumor destruction NOS, photodynamic therapy, electrocautery, cryosurgery, laser, local tumor excision NOS, polypectomy, excisional biopsy, laser excision, partial surgical removal, total surgical removal, debulking surgery, radical surgery, and surgery NOS.

All preliminary analyses, statistical analyses, and Kaplan–Meier curves were generated using IBM SPSS Statistics. Forest plots were generated using GraphPad Prism software. Creighton University Institutional Review Board (IRB) has determined that this study only includes deidentified patient data, deeming it exempt from IRB approval.

## Results

Of the 68,153 patients included, 19.4% were less than 50 years old, 32% were between 50 and 64 years old, 20.5% were between 65 and 74 years old, and 28.1% were greater than 75 years old (Table 1). The median age was 64 years. The majority of patients were White (88.4%) and non-Hispanic (92.0%).

**Table 1:** Demographic frequencies of VSCC

	Variable	Number of patients	Percentage
Age (Median = 64 years old)	< 50 years old	13,240	19.40%
	50–64 years old	21,780	32.00%
	65–74 years old	13,999	20.50%
	≥ 75 years old	19,134	28.10%
Race	White	60,227	88.40%
	Black	5,943	8.70%
	American Indian	205	0.30%
	Asian	571	0.80%
	Unknown	1,207	1.80%
Ethnicity	Non-Hispanic	62,694	92.00%
	Hispanic	2,580	3.80%
	Unknown	2,879	4.20%

Next, overall survival was assessed using Kaplan–Meier survival with log-rank pairwise comparisons. Table 2 demonstrates the mean survival time, which is notably lower in patients 65–74 years old compared to patients 50–64 years old (107.3 months and 152.1 months; *p* < 0.001, respectively).

**Table 2:** KM survival time VSCC

Kaplan–Meier survival time	Variable	Mean survival (Months)	Standard error	95% confidence interval
Age	50–64 years old	152.1	0.84	150.4–153.7
	< 50 years old	***184.9	0.872	183.2–186.7
	65–74 years old	***107.3	0.964	105.4–109.2
	≥ 75 years old	***57.7	0.501	56.7–58.6

The 50–64-year-old age group functions as the reference variable

***indicates *p*-value < 0.001

**Fig. 1:**
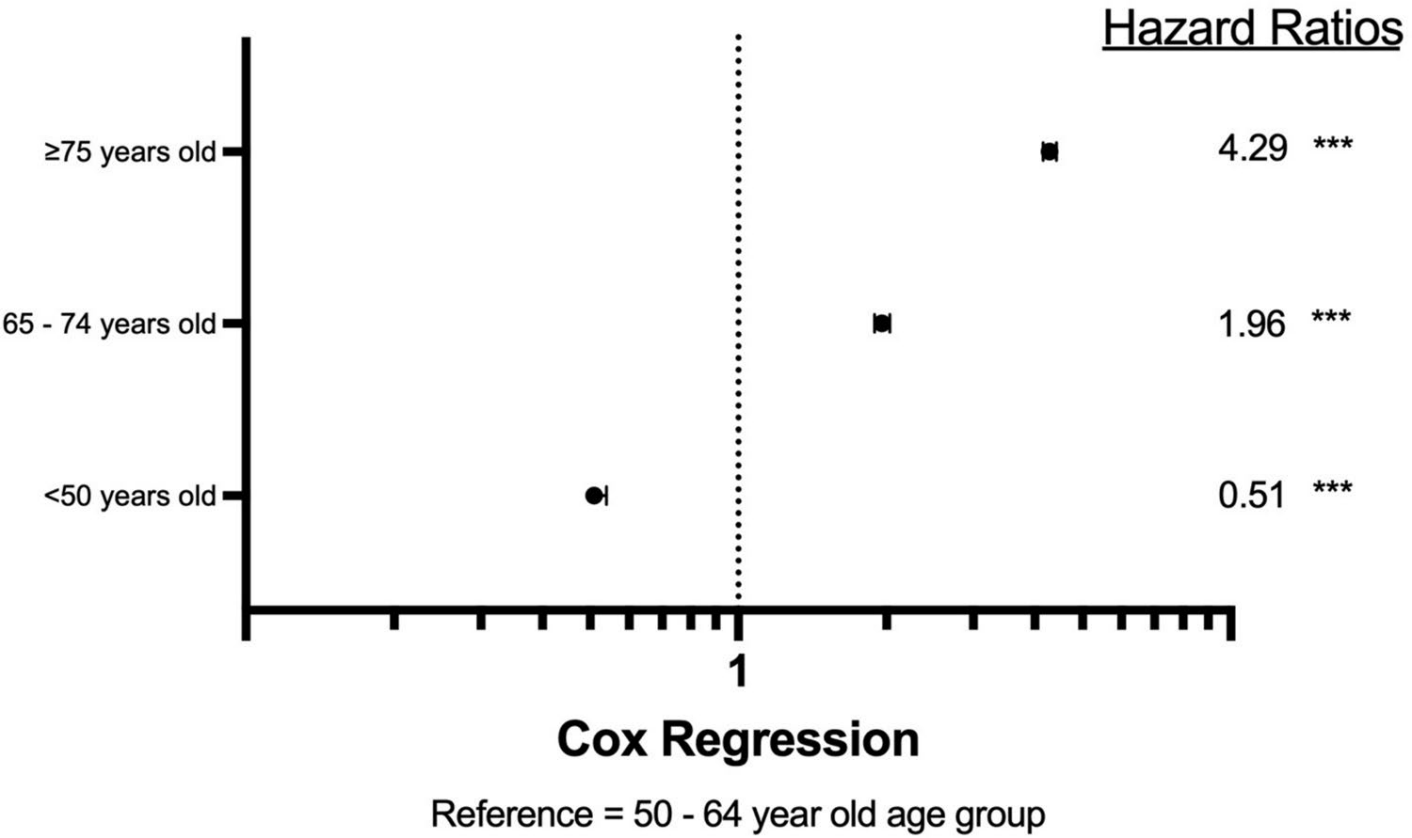
Hazard ratio of VSCC according to age group. Reference group is 50–64-year-olds. *** indicates *p*-value < 0.001

To further investigate the survival differences, hazard ratios were calculated via Cox regression, using the 50–64-year-old age group used as the reference variable. Notably, the 65–74-year-old age group had a hazard ratio of 1.96 compared to the 50–64-year-old age group, indicating 96% greater odds of mortality (*p* < 0.001) (Fig. 1).

**Fig. 2:**
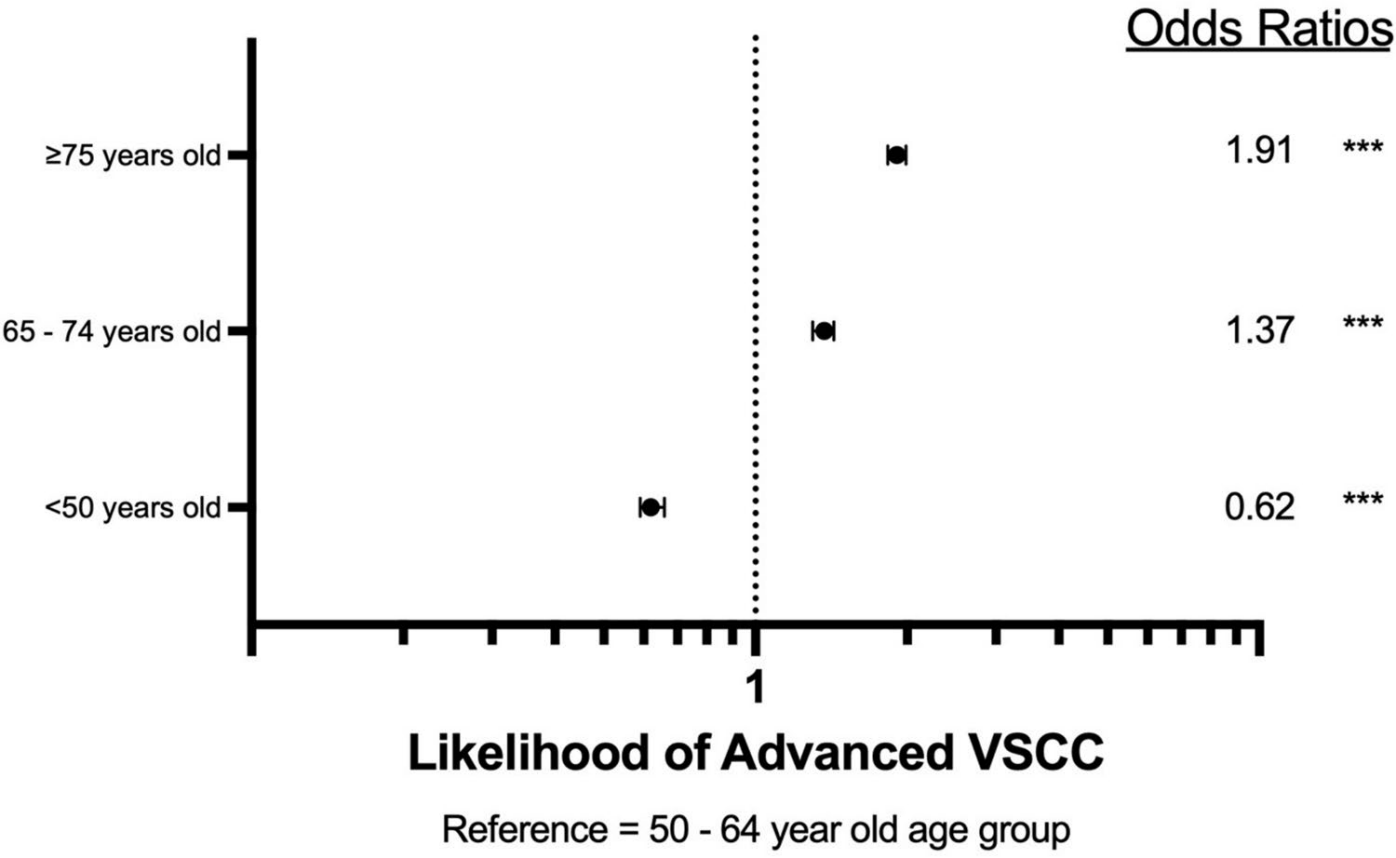
Likelihood of advanced VSCC. Reference group is 50–64-year-olds. *** indicates *p*-value < 0.001

Given the survival discrepancy seen on Kaplan–Meier overall survival and Cox regression, attention was focused on analysis of significant prognostic variables, cancer stage, and surgery. Firstly, binomial regression of advanced VSCC was assessed. The test found that the 65–74-year-old age group had a 37% greater likelihood of having advanced VSCC than those 50–64 years old (*p* < 0.001) (Fig. 2). The cohort of 75 years and older had a 91% (*p* < 0.001) greater likelihood of advanced VSCC than the 50–64-year-old cohort.

**Fig. 3:**
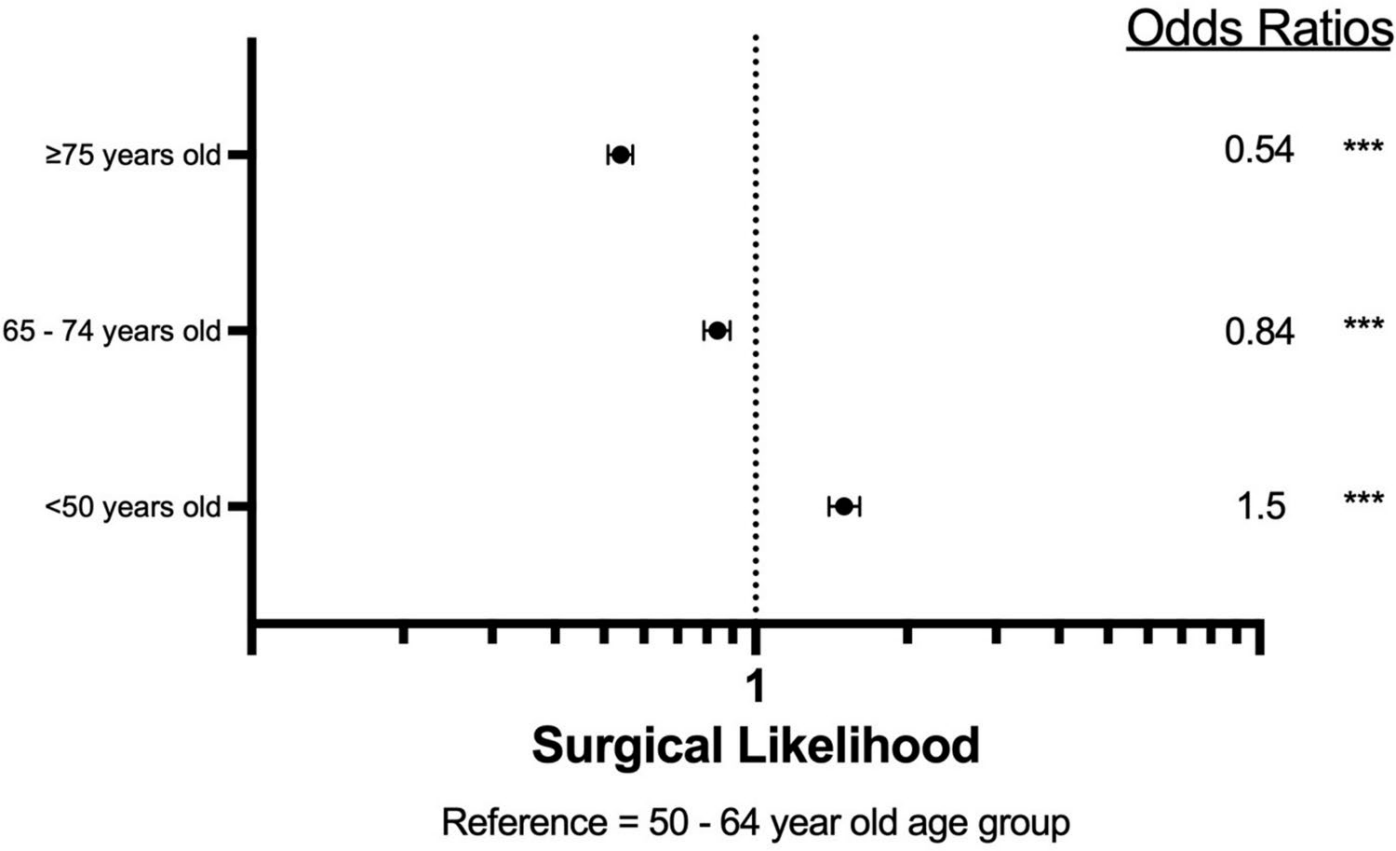
Likelihood of surgery. Reference group is 50–64-year-olds. *** indicates *p*-value < 0.001

In a preliminary univariate Cox regression of the surgery variable, VSCC patients who undergo surgical intervention demonstrated a hazard ratio of 0.293 when compared to those with no surgical intervention. In the multivariate binomial regression of likelihood of undergoing surgery, patients aged 65–74 had 16% lower likelihood of undergoing surgical intervention compared to patients aged 50–64 years old (*p* < 0.001). The 75 years and older groups were 46% less likely to have surgical intervention than the 50–64-year-old group (*p* < 0.001) (Fig. 3).

## Discussion

This study analyzed the data from the NCDB to evaluate if current gynecologic screening guidelines adequately address the disproportionate burden of VSCC on older patients, particularly in patients above the cervical cancer screening age. Currently, there are no formal screening tests for vulvar cancer, and recommendations may fall to external genitalia examinations during cervical cancer screening, which ends at age 65 with negative prior testing [5, 8]. For the purpose of this discussion, the 50–64-year-old cohort is considered *within the screening group*, and the 65–74-year-old cohort is considered the *post-screening group*.

Firstly, the *within screening cohort* was found to have the highest frequency of VSCC (Table 1). Although the age group with the highest frequency varies in previous literature, it is established that VSCC is most commonly a cancer of the older gynecologic patient population. Consistent with previous studies, this analysis also demonstrated that non-Hispanic White patients had the highest frequency and incidence of VSCC cases (Table 2) [1, 3, 9].

Mean survival time decreased as age increased, a trend that aligns with prior studies and demonstrates worse outcomes for older patients [10, 11]. Furthermore, hazard ratios revealed that the *post-screening cohort* had a significantly higher likelihood of death when compared to the *within screening cohort* (Fig. 1). This finding concurs with previous studies which found a higher hazard ratio among older patients, suggesting that other factors may be influencing detection and management of VSCC [11]. However, it is possible that lead-time bias contributed to the observed differences in survival.

One such influence may be the lack of dedicated screening protocols for vulvar cancer, as prior research has suggested methods to improve screening and early detection [12, 13]. Bornstein et al. suggested that the World Health Organization (WHO) expand their gynecologic screening protocols to include vulvar cancer [12]. Geisler and Ganz proposed annual external genitalia examinations as a method for secondary prevention [13]. In the absence of direct screening suggestions, healthcare societies rely on the patient’s knowledge and understanding of their health to seek care when experiencing symptoms of VSCC. However, vulvar cancers have been shown to possess one of the longest delays from symptom onset to the time of diagnosis of gynecologic cancers [12–15]. This may be in part due to the social stigma of the common symptoms of VSCC or symptoms overlapping with benign conditions, such as itching, vaginal discharge, dysuria, and ulceration [12, 16, 17]. Other studies suggest older women are less likely to perform self-examinations to identify a concerning lesion that may prompt an examination [13, 18]. Additionally, reliance on patient healthcare literacy may create survival disparities among patients with lower socioeconomic status [13]. Each of these factors may delay care, as shown by the *post-screening group* possessing a higher likelihood of advanced stage VSCC in this study and others (Fig. 2) [18]. Later-stage VSCC is associated with a higher risk of mortality [19]. Screening is shown to be an effective method to reduce mortality, as cervical cancer screening has drastically decreased the burden of cervical cancer in developed countries [20]. Consistent recommendations, after cervical cancer screening ends, from healthcare societies and colleges may offer clarity in VSCC management, such as periodic external genitalia screenings. The inclusion of routine external genitalia examinations in well woman visits may assist in the early detection of VSCC and broader management options for all patients, as this analysis found that the post-screening cohort was less likely to undergo surgical management (Fig. 3). Other studies have revealed similar findings [11]. Surgical management of VSCC has been shown to be effective, especially in the early stages [21, 22]. However, some studies indicate that primary radiation therapy may demonstrate similar efficacy in overall survival, though with well-documented long-term adverse effects that are detrimental to quality of life [21, 23]. While management is influenced by other factors such as comorbidities, early detection may allow for more options in management and decrease mortality.

The limitations of this study include the limited representation by the NCDB for VSCC patients, as data is collected from accredited hospitals. Additionally, all-cause mortality is utilized for NCDB records. Other limitations include incomplete information from patients that may not have utilized routine screening examinations prior to age 65 and were diagnosed with VSCC. The majority of this study’s population was White, which may indicate other races not utilizing care at NCDB participating hospitals and skewing data analysis.

## Conclusion

Without direct recommendations for VSCC screening, patients older than the cervical cancer screening age group (> 65 years) may experience worse outcomes and advanced stages of VSCC. Establishing consistent guidelines regarding vulvar cancer screening may assist in prompt detection of VSCC, allowing for early stages to be diagnosed, more treatment options, and less treatment morbidity and mortality in the *post-screening* gynecologic patient population.

## Data Availability

Data are publicly available from the National Cancer Database found at https://www.facs.org/quality-programs/cancer-programs/national-cancer-database/
